# Graphene Oxide Paper Manipulation of Micro-Reactor Drops

**DOI:** 10.3390/mi14071306

**Published:** 2023-06-26

**Authors:** Zhixiong Song, Eric Shen Lin, Md Hemayet Uddin, Hassan Ali Abid, Jian Wern Ong, Tuck Wah Ng

**Affiliations:** 1 Laboratory for Optics and Applied Mechanics, Department of Mechanical & Aerospace Engineering, Monash University, Clayton, VIC 3800, Australia; zhixiong.song@monash.edu (Z.S.); eric.lin@monash.edu (E.S.L.);; 2 Melbourne Centre for Nanofabrication, 151 Wellington Rd., Clayton, VIC 3168, Australia; hemayet.uddin@monash.edu

**Keywords:** digital microfluidics, graphene oxide, droplets, self-wrapping, self-folding

## Abstract

Digital microfluidics, which relies on the movement of drops, is relatively immune to clogging problems, making it suited for micro-reactor applications. Here, graphene oxide paper of 100 μm thickness, fabricated by blade coating sedimented dispersions onto roughened substrates, followed by drying and mechanical exfoliation, was found to be relatively free of cracks and curling. It also exhibited high wettability and elasto-capillary characteristics. Possessing low enough stiffness, it could rapidly and totally self-wrap water drops of 20 μL volume placed 2 mm from its edge when oriented between 0 and 60° to the horizontal. This complete wrapping behavior allowed drops to be translated via movement of the paper over long distances without dislodgement notwithstanding accelerations and decelerations. An amount of 2 drops that were wrapped with separate papers, when collided with each other at speeds up to 0.64 m/s, were found to eschew coalescence. This portends the development of robust digital microfluidic approaches for micro-reactors.

## 1. Introduction

Microfluidics technology continues to be widely researched and developed to handle and manipulate microliter volumes of fluids on substrates. It has been usefully applied in a variety of biochemical tests [1,2,3] as well as micro-reactor applications [4,5,6], offering the ability to minimize material/reagent consumption as well as waste. Microfluidics can broadly be accomplished as either channel-based or digital microfluidics. Channel-based microfluidics suffer from clogging issues due to the narrow sizes of the channels [7,8]. Digital microfluidics, which relies on the movement of drops, is, alternatively, relatively more immune to this problem [9,10,11,12]. One of the exciting areas of application of digital microfluidics is in the creation of micro-reactors [13,14,15]. The field has also been extended by the use of liquid marbles instead of drops. Liquids marbles comprise a coating of particles around the liquid drop, imbuing unique functionalities when used in conjunction with digital microfluidics [16,17,18]. Despite the relative attractiveness of digital microfluidics, the drop’s movement over surfaces using electrowetting on dielectrics (EWOD) is prone to material adsorption issues [19]. This may be overcome somewhat by applying complicated functionalization of the EWOD to reduce the degree of adsorption while still allowing the drop to maintain a suitable extent of contact angle hysteresis [20].

The use of paper as the substrate for microfluidics had been advocated due its low cost, lightweight, availability in a wide range of thicknesses, as well as its ease of stacking and storing features [21]. Paper substrates have also been utilized in the creation of digital microfluidic devices [22,23]. It should be noted that the intrinsic high wettability of paper (due to its fibrous porosity) can be a hinderance in many digital microfluidic operations. For this reason, other types of substrates, which have the form of paper but not its strong wetting characteristics, have been investigated for digital microfluidics [24,25,26].

Graphene oxide (GO) is a vital two-dimensional precursor in the manufacture of graphene. It is now, due to its relative lower cost and ease in preparation, finding increasing usage in many important applications [27,28,29]. Free standing GO or GO paper was first developed using a flow-directed assembly process of individual graphene oxide sheets [30]. It has since been used for novel applications such as dehumidification [31] and the creation of flexible terahertz devices [32].

When thin elastic sheets come in contact with liquid drops, there is a competition between surface tension and elasticity [33,34]. The resultant deformation of the sheet can be extreme, to the extent that it may completely encapsulate the liquid body.

In this work, we report a method that uses the blade coating of sedimented GO dispersions on roughened PVC substrates, followed by drying and mechanical exfoliation to create GO paper. Its microstructure, thickness change from evaporation, wettability, as well as elasto-capillary and liquid self-wrapping characteristics are examined. Additionally, its potential usefulness in the robust transfer of drops with immunity from interactions in digital microfluidics is also illustrated.

## 2. Materials and Methods

### 2.1. GO Paper Preparation

In total, 1 g of graphite powder (Sigma (Burlington, MA, USA), 282863) and 0.5 g of sodium nitrate (Sigma, S5022) were first mixed together in a beaker. A total of 23 mL of sulphuric acid (Sigma, 339741) was added to the mixture with constant stirring. After 1 h, 3 g of potassium permanganate (Sigma, 223468) was gradually added (to ensure that overheating did not occur) to the solution. The solution was then stirred for 12 h, and then, diluted with 500 mL of water followed by vigorous stirring. A total of 5 mL of 30% hydrogen peroxide (Sigma, 223468) was added to ensure completion of the reaction with potassium permanganate. The resulting mixture was then washed with hydrochloric acid (Sigma, 295426) and deionized water, followed by filtration and drying to produce a suspension of GO sheets.

GO sedimentation was achieved by placing 400 mL of the GO suspension in a cylindrical beaker and allowing it to stand for up to 480 h. The progress was periodically inspected with images recorded. When sedimentation was sufficiently visible, the fraction of sedimented volume was determined by taking the ratio of sedimentation height against the total (sedimentation + supernatant) height of the beaker contents.

Polyvinyl chloride (PVC) substrates were used as templates to facilitate coating creation of the GO paper. They were cleaned in absolute alcohol and allowed to dry in air for 10 min before being roughened (service provided by Dextech Technologies Pty Ltd. (Brandon Park, VIC, Australia)) to high degrees of uniformity. After cleaning the roughened surfaces in running water for 2 min, they were placed in a vacuum drying oven (Si Yang (Suqian, China), SGZX-6020) under a 40 °C temperature setting for 5 min. The PVC substrates were ready to be used for the coating treatment process after their removal from the oven.

Specific volumes (1.7 mL) of the sedimented GO solution were aspirated using a pipette and deposited on the PVC substrate. A plastic “blade” positioned 2.1 mm above the substrate was moved across the GO solution at a constant speed (0.8 cm/s) to uniformly spread it out across the substrate. The blade-coated GO substrates were then transferred to a vacuum drying oven (Si Yang, SGZX-6020) and kept in it for a duration of 24 h at a setting temperature of 40 °C. Upon removal from the oven, the evaporated GO solution existed as a thin sheet on the substrate. They could be readily peeled off using tweezers to form the GO paper. The papers were placed in airtight sample containers to avoid any contamination while awaiting to be used.

### 2.2. Thickness Characterization

The thickness of the GO paper was assessed using an optical profilometer (Bruker Contour GT-I (Billerica, MA, USA)). Prior to measurement, they were stored in a dust-free container to minimize particulate contamination from the environment. Processing and analysis were performed on the instrument’s accompanying software (Contour Elite (Madison, WI, USA)).

### 2.3. Scanning Electron Microscopy Characterization

The paper sheets substrates were mounted onto aluminum stubs and placed inside the scanning electron microscope (NOVA (Rehovot, Israel), Model NovaSEM450) for imaging. The samples were subjected to a vacuum pressure of 7 × 10^−3^ Pa prior to imaging at an accelerating voltage of 5 kV and spot size of 2.5. Thin carbon coatings of 2 nm were applied to the samples to enhance their conductivity to alleviate the charging issues to the best extent. Micrographs were then obtained at different magnifications.

### 2.4. Wettability Characterization

Fifty µL deionized water was dispensed on each of the substrates that contained the thin GO coating (i.e., not peeled off) on its surface. Top view images were recorded under 500 lux of low glare and shadow free lighting (as prescribed in the EN 12464-1 standard) in order to examine their water spreading characteristics. A power law was applied to relate the average radii of the main contact line *R_m_* and capillary contact line *R_c_* determined at various times *t* using

(1) $$R_{m}=kt^{a}$$

In Equation (1), the value of *a* was determined using the slope of the *R_m_* and *R_c_* versus *t* trends in logarithmic scale. With this value, *k* was then ascertained via the slope in the *R_m_* and *R_c_* versus *t* trends (this time not in logarithmic scale).

### 2.5. Elasto-Capillary Wrapping Behavior

**Figure 1:**
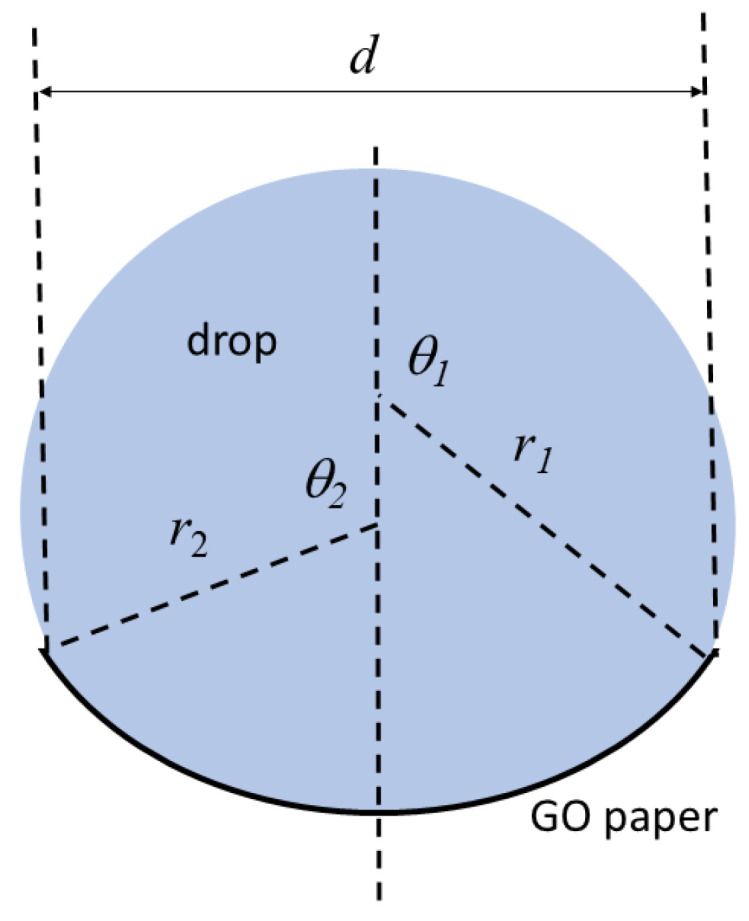
The geometry of an elastic sheet that is deformed by the capillary forces of a drop.

The GO papers (peeled off from the roughened PVC substrate) were cut into various sizes with a constant width of 3.2 mm and lengths ranging from 4.5 mm to 9 mm. They were placed horizontally on a flat surface as drops of water of 20 µL were placed at the center of each paper and their wrapping process recorded via a digital microscope CCD camera (Koolertron 5 MP 20–300× (Hong Kong, China)). The resulting wrapping images were analyzed by software (ImageJ) to determine the critical values shown in Figure 1. From these values, a metric s that depicts the drop volume effect in each case could be calculated using [34]

(2) $$s=r_{1}{}^{2}(\theta_{1}-sin\theta_{1}cos\theta_{1})+r_{2}{}^{2}(\theta_{2}-sin\theta_{2}cos\theta_{2})$$

A plot of the *d* against *s* values was created to interrogate the elasto-capillary characteristic in the GO paper.

### 2.6. Edge Wrapping Behavior

**Figure 2:**
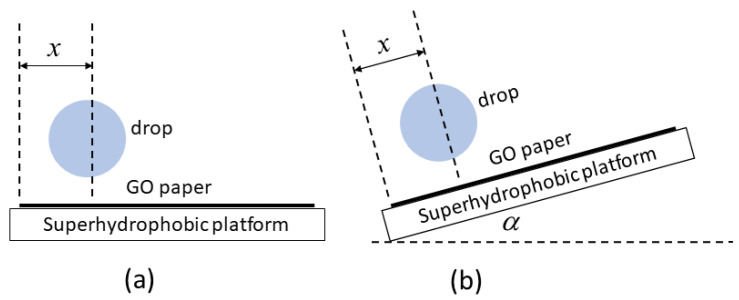
Experiments conducted to investigate the edge wrapping behavior of GO paper in which (**a**) the paper is located horizontally on a superhydrophobic platform and on which a water drop is dispensed at various distances *x* from the edge, as well as when (**b**) the paper is located on a superhydrophobic platform at various inclination angles *α* to the horizontal and a water drop is dispensed at a fixed distance *x* from the edge.

The GO papers of a rectangular size of 17.4 mm × 3.2 mm were placed on a horizontal platform superhydrophobic surface (to prevent any preferential adhesion of liquid to it) as depicted in Figure 2a. Drops with a volume of 20 µL were deposited on each of the papers with variable distances from the drop center towards the paper width side. Side images were recorded using a digital microscope CCD camera (Koolertron 5 MP 20–300×) and their shape characteristics analyzed using an imaging software (ImageJ).

Another set of images were now obtained with the GO paper positioned at different inclination angles to the horizontal (guided using the more rigid superhydrophobic platform) in which drops with a volume of 20 µL were dispensed at a fixed distance of 1.8 mm from the edge (see Figure 2b). Side images were again recorded and analyzed as previous.

### 2.7. Transport and Interaction of Drops Wrapped in GO Paper

In one set of experiments, water drops of 20 µL volume were dispensed at a fixed distance of 1.8 mm from the edge of a piece of GO paper with a size of 17.4 mm × 3.2 mm rested on a superhydrophobic platform that was placed horizontally. The GO paper, with the drop wrapped at its edge, was translated at various speeds across the superhydrophobic platform. Video images were recorded to ascertain its transport stability.

In another set of experiments, water drops of 20 µL volume were dispensed at a fixed distance of 1.8 mm from the edges of 2 GO papers with a size of 17.4 mm × 3.2 mm placed on a superhydrophobic platform placed horizontally. One drop contained red food dye while the other had blue food dye to distinguish them from each other. The GO papers, with the drop wrapped at their respective edges, were moved towards each other across the superhydrophobic platform to contact each other. Video images were recorded to ascertain if any interaction between the liquid samples occurred.

## 3. Results and Discussion

**Figure 3:**
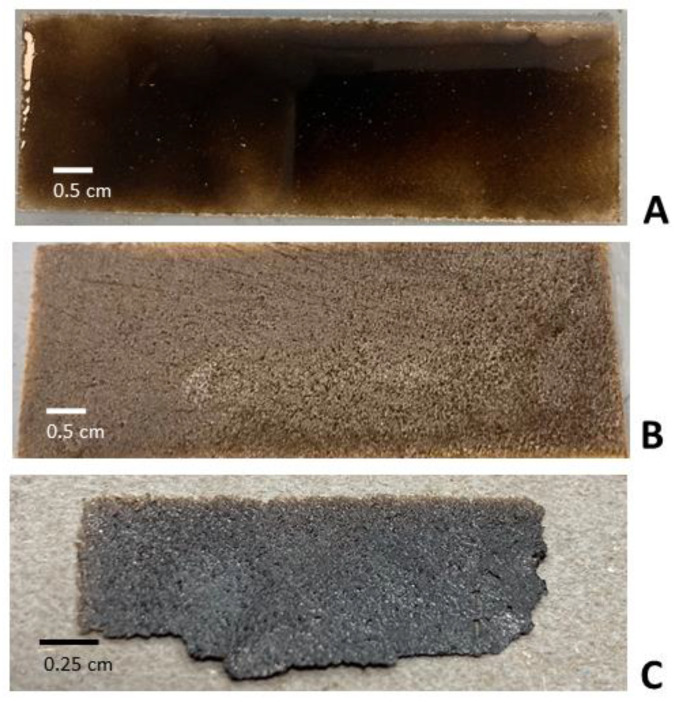
The GO solution spread by blade coating (**A**) significantly reduced in thickness after drying (**B**). The mechanically exfoliated GO paper was found to be highly flattened (**C**).

The sedimented GO solution was found to possess viscous or viscoelastic characteristics. Two methods, spin coating and blade coating, were explored to try to spread it evenly on the PVC substrate. It was found that only blade coating was feasible to do so (see Figure 3A). It has been previously established that blade coating offers the advantages of little material waste and well-defined coating [35]. It also allows for subsequent quick direct drying in the oven without the need of any solvent annealing [36].

After drying in the oven, the thickness of this deposit was seen to considerably reduce (Figure 3B). It was found that unless the PVC substrate was roughened prior, it was almost impossible to exfoliate out the paper. This is due to the peeling force being modulated by the surface roughness of the substrate [37]. The GO paper, when peeled from the roughened PVC substrate, was found to be highly flattened with little extents of curling (see Figure 3C).

**Figure 4:**
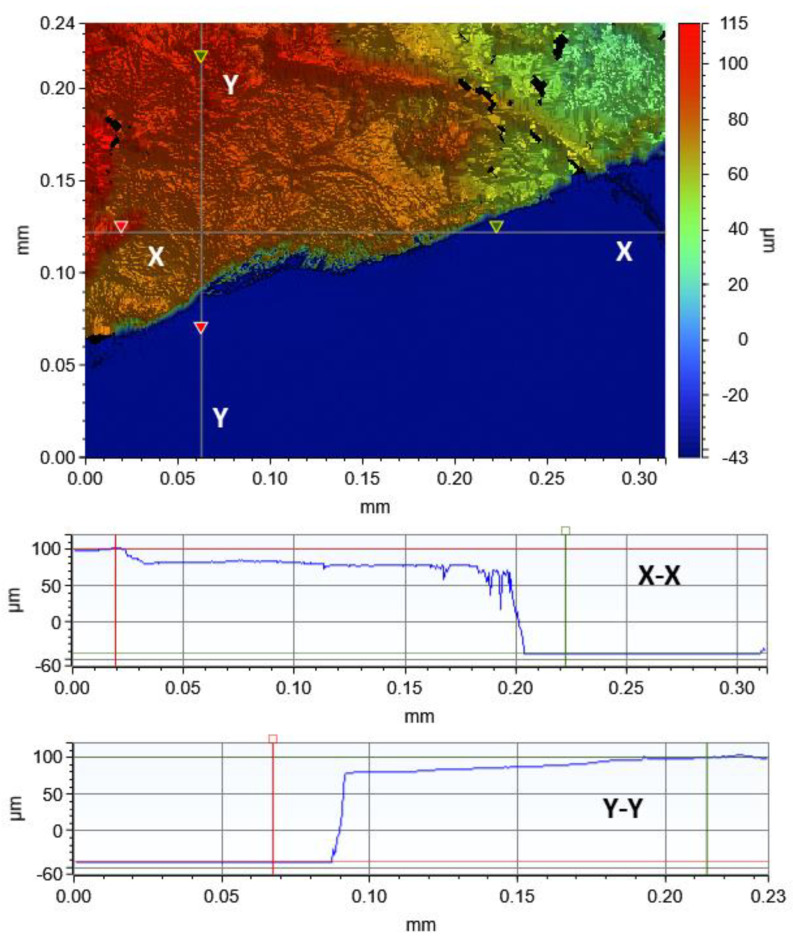
Typical optical profilometry trace of the GO paper with the height distributions in the X-X and Y-Y sections included.

The test to determine the thickness of the GO paper was done by first resting it on top of a flat substrate. From a series of optical profilometry traces made near the boundary (between the GO paper and substrate), and height profiles derived from them (see Figure 4), the average thickness, obtained from 5 separate GO paper samples, was determined to be 99.56 μm ± 35.13 μm. This constituted an almost 95.2% loss in its thickness due to liquid (water) evaporating away during drying.

The profilometry scans also showed low degrees of cracking with almost no curling of the GO paper notwithstanding the significant thickness reduction that it underwent during drying. As the sedimented GO solution can be considered to be a colloid, the forming films that evolve during drying typically results in increasing stress gradients that can result in cracking [38]. Curling basically forms as a result of several main cracks appearing and interacting on the film. That the GO paper is generally devoid of cracking and curling imputes low degrees of stress gradients developing as the GO paper forms from the drying colloidal GO solution. Such a behavior had been previously investigated with capillary dispersions (ternary mixtures of solid particles immiscible fluids), where stress build-up comes from frustrated shrinkage while stress relief is derived from relaxation due to the action of strong particle networks. Under specific formulations and drying conditions, the latter can dominate to the extent that cracking may be prevented [39].

**Figure 5:**
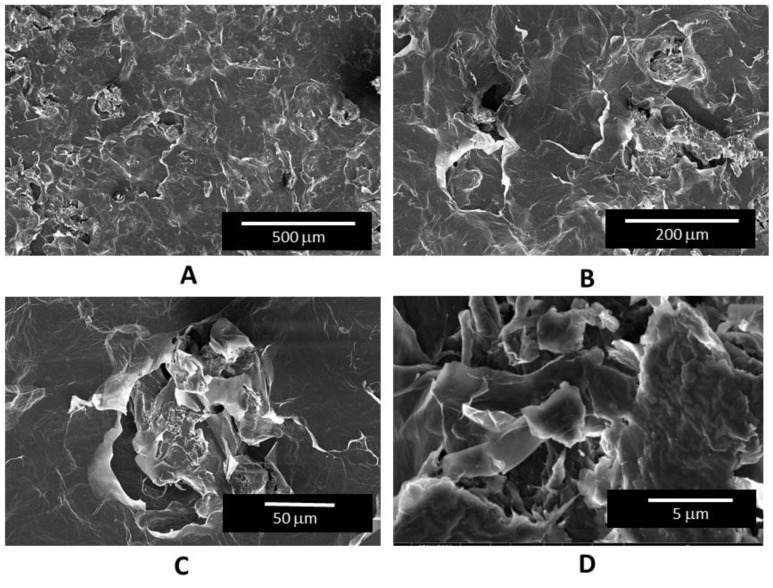
Scanning electron microscopy images of GO paper under the magnification times of (**A**) 250×, (**B**) 650×, (**C**) 1500×, and (**D**) 20,000× magnifications. Under high magnifications, microscopic defects can be seen populating the surface, which are likely template transferred from the roughened PVC substrate.

While the optical profilometry conducted revealed low distributions of cracks on the GO paper, the SEM images, particularly at the higher magnifications, showed a preponderance of microscopic defects on the surface (see Figure 5). These defects were likely the result of a “templating transfer” effect from the roughened PVC substrate. They also appear to only imbue a matted surface appearance on the GO paper, and hence, not expected to affect their application purposes in this work.

**Figure 6:**
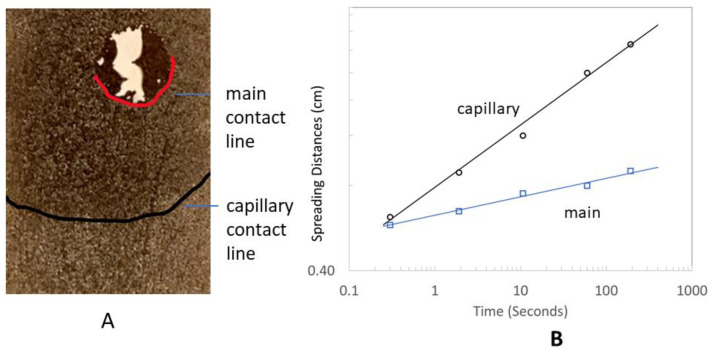
Recorded image (**A**) of water spreading on GO paper which shows the presence of main (red line) and capillary (black) contact lines. The spreading distance versus time of both lines adhere to the power law (**B**).

The wetting characteristics were studied with the dried GO paper unpeeled from the PVC substrate to avoid difficulties in interpretation from warping and other distortions. As the paper was highly wetting (with almost zero contact angles), wettability was characterized by the movement of the capillary and main contact lines when water was dispensed on the surface [40,41] (see Figure 6A). From the graphs of their spreading distance against time, both the main and capillary contact lines were found to adhere to the power law given in Equation (1). From the equation, the values were *a* = 0.065, *k* = 0.628, and *a* = 0.22; *k* = 0.788 for main and capillary contact lines, respectively.

**Figure 7:**
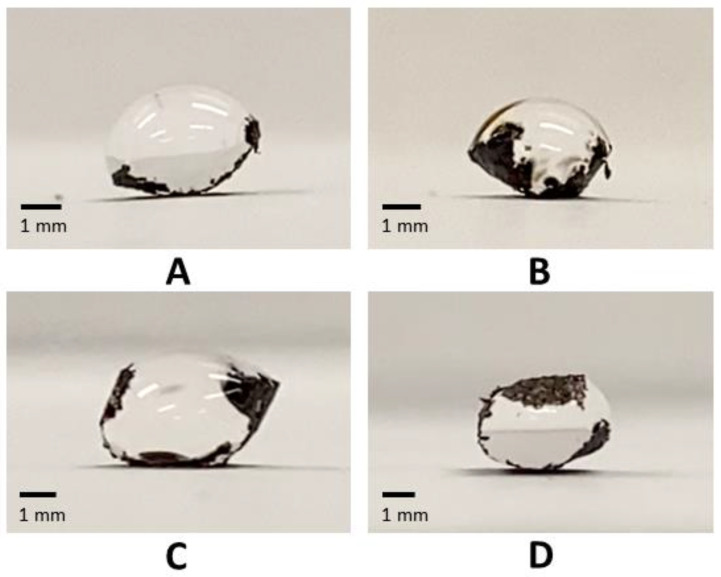
Water drops of 20 μL volume being wrapped by GO paper with lengths of (**A**) 4.5, (**B**) 5.6, (**C**) 6.7, and (**D**) 8.9 mm.

**Figure 8:**
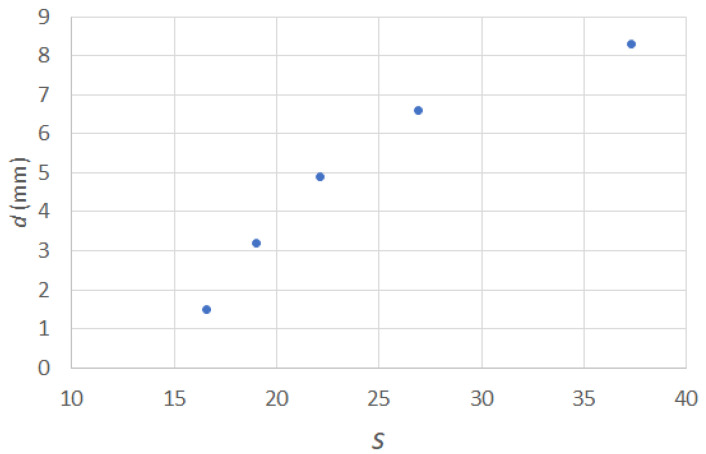
Plots of distance d between tips against the dimensionless drop volumes as described in Equation (2).

In placing water drops at the center of GO paper of various lengths (4.5 to 8.9 mm), the wrapping behavior of the GO paper was clearly evident (see Figure 7). Due to limitations of not being able to deposit the drop exactly at the center of the GO paper, some departures from the idealized behavior, as depicted in Figure 1, can be expected. However, it was still possible to approximate the parameters needed to determine the volume related metric *s* as described in Equation (2). The trend of the *d* against *s* plot, as shown in Figure 8, was consistent with that previously reported [34], confirming the presence of elastic characteristics in the GO paper. Based on the derivations that relate the length of sheet, its bending stiffness, and the surface tension of water (= 0.072 Nm^−1^) [34], the bending stiffness could be approximated as ~10^−7^ Nm. The bending stiffness *B* is in turn related to the Young’s modulus E, Poisson’s ratio *μ*, and thickness *h* via

(3) $$B=\frac{Eh^{3}}{12\left(1-\mu^{2}\right)}$$

Applying values of *μ* = 0.27 [42] and *h* = 0.1 mm, gives a Young’s modulus of ~0.8 MPa. This value is significantly lower than that of monolayer graphene oxide sheets [43] (~200 GPa), indicating that the GO paper created in this manner is very flexible, and hence, suited for applications that require elasto-capillary wrapping.

**Figure 9:**
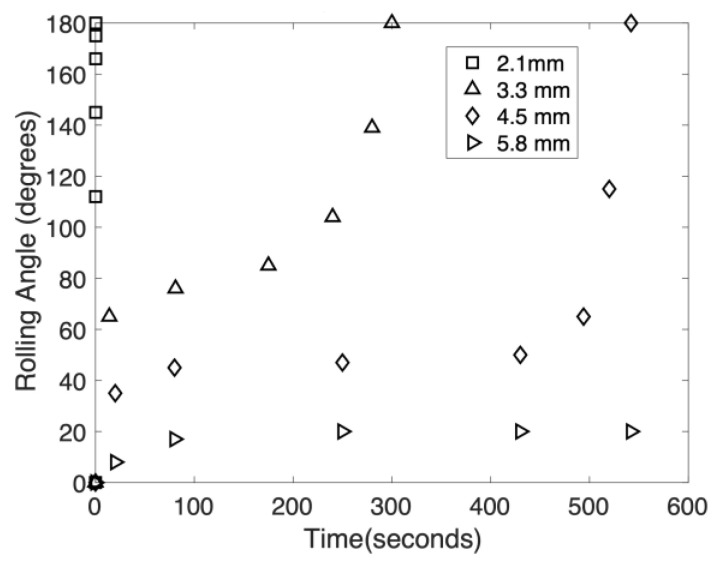
The time evolution of wrapping with GO paper of width of 3.2 mm with a 20 μL water located at distances of 2.1, 3.3, 4.5, and 5.8 mm from 1 end.

**Figure 10:**
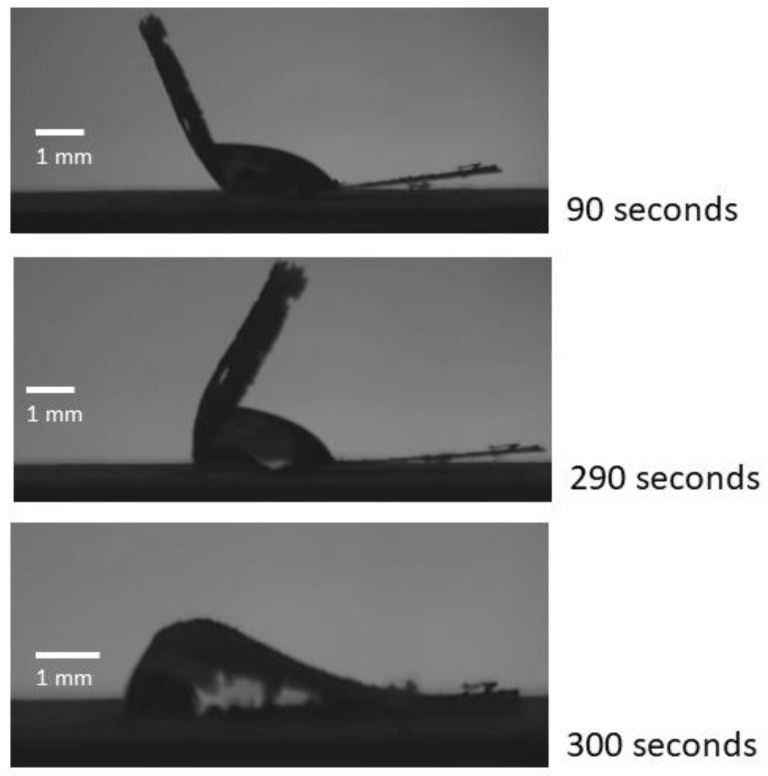
Sequence of images showing how a 20 μL water drop placed 3.3 mm from the edge is gradually wrapped after time lapses of 90, 290, and 300 s.

In practical applications involving micro-reactors using digital microfluidics, the ability to develop elasto-capillary wrapping of drops at one end of the GO paper will allow the opposite end to be used for motion actuation. When the 20 μL water drop was placed 2.1 mm from 1 end, the GO paper wrapped around it fully and almost instantaneously (see Figure 9). As this distance was increased, the time taken also increased. Figure 10 provides image sequences recorded in which complete wrapping was only accomplished after 300 s. It was also discovered that if the drop was placed further than 4.5 mm from the edge, complete wrapping would not occur at all. This behavior as well as the delayed wrapping effect can be attributed to the length of overhanging material (from the drop center to the edge of the GO paper) which introduces a resisting torque to the capillary action that seeks to wrap the paper around the drop. Notwithstanding the nuanced physics behind this, placing the drop as close as possible to the edge will reduce the wrapping time that is typically desired in facilitating digital microfluidics. It should be noted that impact delivery of the drop may be used to hasten the wrapping [44], albeit the trade-off of requiring the necessary equipment and the accuracy needed to do this accurately will likely render this approach rather unfeasible.

**Figure 11:**
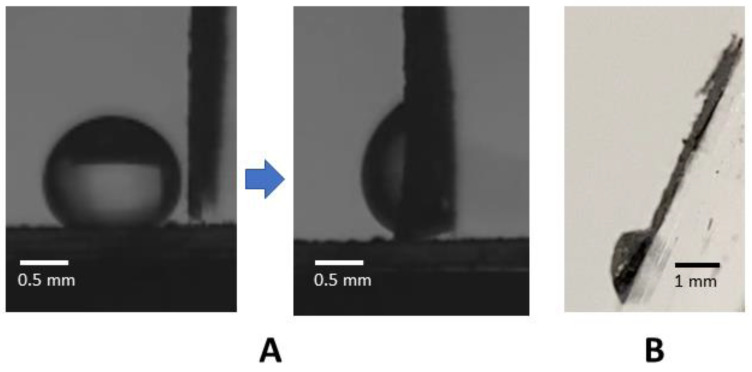
A 20 μL drop placed on a superhydrophobic surface was able to be picked up by the GO paper oriented 90° to the horizontal but without wrapping occurring (**A**). The location of the GO paper at angles not exceeding 60° to the horizontal was able to achieve drop pick-up with wrapping (**B**).

Apart from dispensing the water drop on the GO paper, it might be necessary to pick an already dispensed drop on a strongly hydrophobic surface using the GO paper. With the GO located 90° to the horizontal, wrapping did not occur (Figure 11A). It was, however, found that the drop pick-up with wrapping with the GO paper could be attained by having the paper oriented <60° to the horizontal.

**Figure 12:**
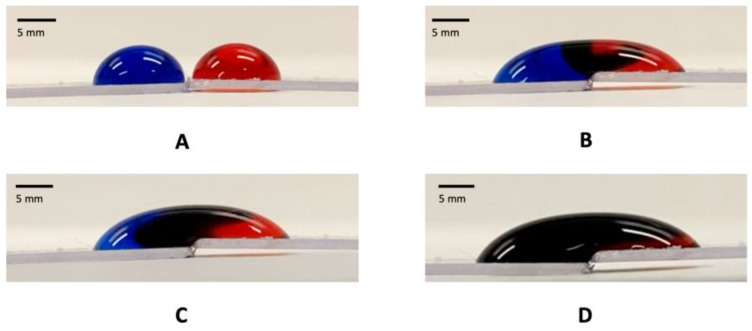
Sequence of images illustrating the color change when 2 drops, initially colored with red and blue dyes, respectively (**A**), interact with each other to yield a mixed purple color 1 s (**B**), 10 s (**C**), and 100 s (**D**) after contacting each other.

In digital microfluidics, it is typical to transfer drops on substrates to undergo various biochemical operations. When the drops are close to each other, there is a possibility for the drops to inadvertently coalescence, and thus, result in unwanted reagent mixing (see illustration in Figure 12). It is also noteworthy that in using EWOD for digital microfluidics, drop transport is typically limited to ~10 mm due to the complexity and cost involved in fabricating the substrates.

**Figure 13:**
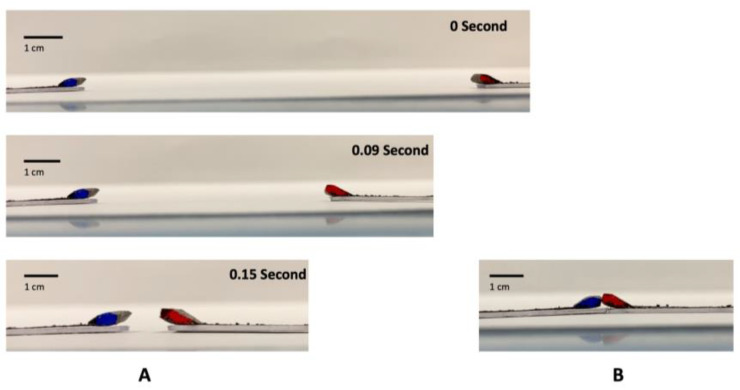
Sequence of images illustrating two drops wrapped at the ends of GO papers being able to be moved rapidly and over long distances (**A**). When they are made to collide with each other with speeds up to 0.64 m/s, the drops do not coalescence with each other (**B**).

When drops are wrapped using GO papers at their distal ends (see Figure 13A), they can be moved over long distances as well as rapidly without being displaced by inertial forces (which typically occur when drops are instead placed on accelerated and decelerated open substrates). More importantly, the local GO paper wrapping also renders the drops immune to coalescence when they interact with each other; even when they were moved towards each other at speeds of up to 0.64 m/s (see Figure 13B), which is more than 60 times faster than digital microfluidic drops typically transferred via EWOD. This is in contrast to liquid marbles, which although relatively immune to coalescence, will still do so when they are moved together at ~0.4 m/s [45]. Liquid marbles are also not as readily transportable as drops are on substrates using EWOD due to their encapsulation by particles.

In summary, a new approach of using highly flexible GO paper to locally wrap drops at its edges for robust transport is presented and demonstrated here. It has the promise of facilitating digital microfluidics for micro-reactor applications.

## Data Availability

This work does not involve the use of datasets. The experimental data may be obtained through the corresponding author.
